# Prognostic significance of cystatin SN associated nomograms in patients with colorectal cancer

**DOI:** 10.18632/oncotarget.23041

**Published:** 2017-12-08

**Authors:** Taiyuan Li, Qiangqiang Xiong, Zhen Zou, Xiong Lei, Qunguang Jiang, Dongning Liu

**Affiliations:** ^1^ Department of General Surgery, The First Affiliated Hospital of Nanchang University, Nanchang, Jiangxi 330006, China

**Keywords:** CST1, immunohistochemistry, nomograms, prognosis, colorectal cancer

## Abstract

Colorectal cancer (CRC) is one of most malignant tumors, mainly due to its high rate of metastasis and recurrence. The prognosis of CRC is difficult due to early CRC patients have no specific symptoms. Therefore, it is emergent to identify a biomarker for CRC prognosis. Cystatin SN (CST1) shows elevated expression in many tumors, but its role in CRCs is still unknown. Through immunohistochemistry analysis, we found that CST1 was upregulated in CRC samples. The survival analysis had demonstrated that high CST1 expression was closely associated with poor clinical status, providing that CST1 plays a role in CRC tumorigenesis. Furthermore, nomograms were generated using CST1 levels and other factors to evaluate survival of CRCs. We evaluated the reliabilities of these nomograms using an independent cohort of 141 CRC cases and found that high CST1 expression is linked to low survival, which is consistent with the clinical results. Thus, we could predict the survival of a CRC patient via these nomograms. In addition, the multivariate analysis identified CST1 as an independent prognostic factor for CRCs, providing CST1 as a biomarker for CRC prognosis. Taken together, our studies revealed a close relationship between CST1 and CRCs, suggesting that CST1 possibly acts as a marker for CRC prognosis and a target for CRC therapy.

## INTRODUCTION

Colorectal cancer (CRC) ranks as the fifth-most common cancer and the most common cause of tumor-related mortality worldwide [1], mainly owing to its high rate of metastasis and recurrence. Despite increasing studies have improved the survival of CRC patients, a subset of patients will develop local recurrences and metachronous metastases after resection of the primary tumor [2, 3]. The current American Joint Committee on Cancer (AJCC) TNM staging system is widely used as a guideline for staging and survival estimates [4]. However, many different clinical outcomes have been reported in patients with the same stage and similar treatment regimens. To properly address postoperative surveillance and treatment, it is fruitful to explore prognostic biomarkers to characterize the heterogeneity of CRCs [2, 5, 6]. However, most biomarkers are unfit to be adopted in clinic, possibly due to the lack of reproducibility and/or standardization [2, 6–8]. Therefore, a better understanding of the biochemical signaling pathways involved in CRCs potentially aid to identify valuable prognostic biomarkers, culminating in improving the prognoses and providing appropriate clinical treatment for CRC patients.

Adjuvant chemotherapy is accepted as a standard component of therapies for patients with stage III CRCs and significantly improves their outcomes [9]. A large proportion of stage II patients are cured by surgery alone, but perforation of the tumor and an insufficient number of examined lymph nodes are related to inferior outcome, so adjuvant chemotherapy is generally used for these patients [10]. A part of stage II patients without increased recurrence risk based on current clinical factors still evolve relapse. However, the effects of adjuvant chemotherapy on stage II patients are complex and may even be harmful for some patients [9–11]. Thus, it is urgent to find a new biomarker for CRC diagnosis, especially for stage II CRCs.

Cysteine proteases, such as cathepsins and papain, are proteolytic enzymes that are expressed widely in tissues and play roles in many cellular processes, including inflammatory tissue destruction, immune response, tissue remodeling, and cell migration [12–15]. Previous clinical studies have demonstrated that the expressions of cysteine proteases are increased in various malignant tumors, while inhibitions of cysteine proteases attenuate tumor growth and metastasis [16]. The cystatin (CST) family comprises proteins that specifically inhibit the proteolytic activities of cysteine proteases [17, 18]. Cystatin SN (CST1), a kind of CST proteins, plays a considerable role in the processes of inflammation and tumorigenesis [19, 20]. Other group also reveals that CST1 is essential for regulating cysteine protease activities and closely links to gastric tumorigenesis through T cell factor-mediated proliferative signaling [21]. CST1 possibly contributes to the proliferation of cancer cells and acts as a potential biomarker for the early diagnosis of pancreatic cancers [21, 22]. Furthermore, in non-small cell lung cancers (NSCLCs), patients with high CST1 expression show more serious recurrence/metastasis and poorer survival compared to low CST1 expressed counterparts [23]. Contradictorily, CST1 shows higher expression in peritumoral tissues than in the esophageal squamous cell carcinoma (ESCC) tissues [24]. Compared to ESCC patients with low levels of CST1, high expression patients have more favourable survivals after surgical treatment [24]. On the other hand, many kinds of CRC cells express elevated CST1, indicating that CST1 possibly plays some roles in CRC tumorigenesis [25]. However, the relationship between CST1 and CRCs are still unclear. Therefore, dissection the links of CST1 and CRCs is valuable for CRC diagnosis and subsequent clinical treatment.

In this study, through immunohistochemistry assays, we found that CRC tissues showed high CST1 protein. We further demonstrated that closely associations existed between CST1 levels and clinicopathological factors, providing CST1 as a potential target for CRC diagnosis and clinical treatment. In addition, we generated two predictive nomograms integrating CST1 expression, the level of CEA, tumor grade, tumor depth, and lymph node metastasis to assess the risk score for overall survival (OS) and disease-free survival (DFS) of CRC patients. Taken together, our studies will pave a way for CRC diagnosis and therapy.

## RESULTS

### CST1 is overexpressed in CRC tissues

**Figure 1:**
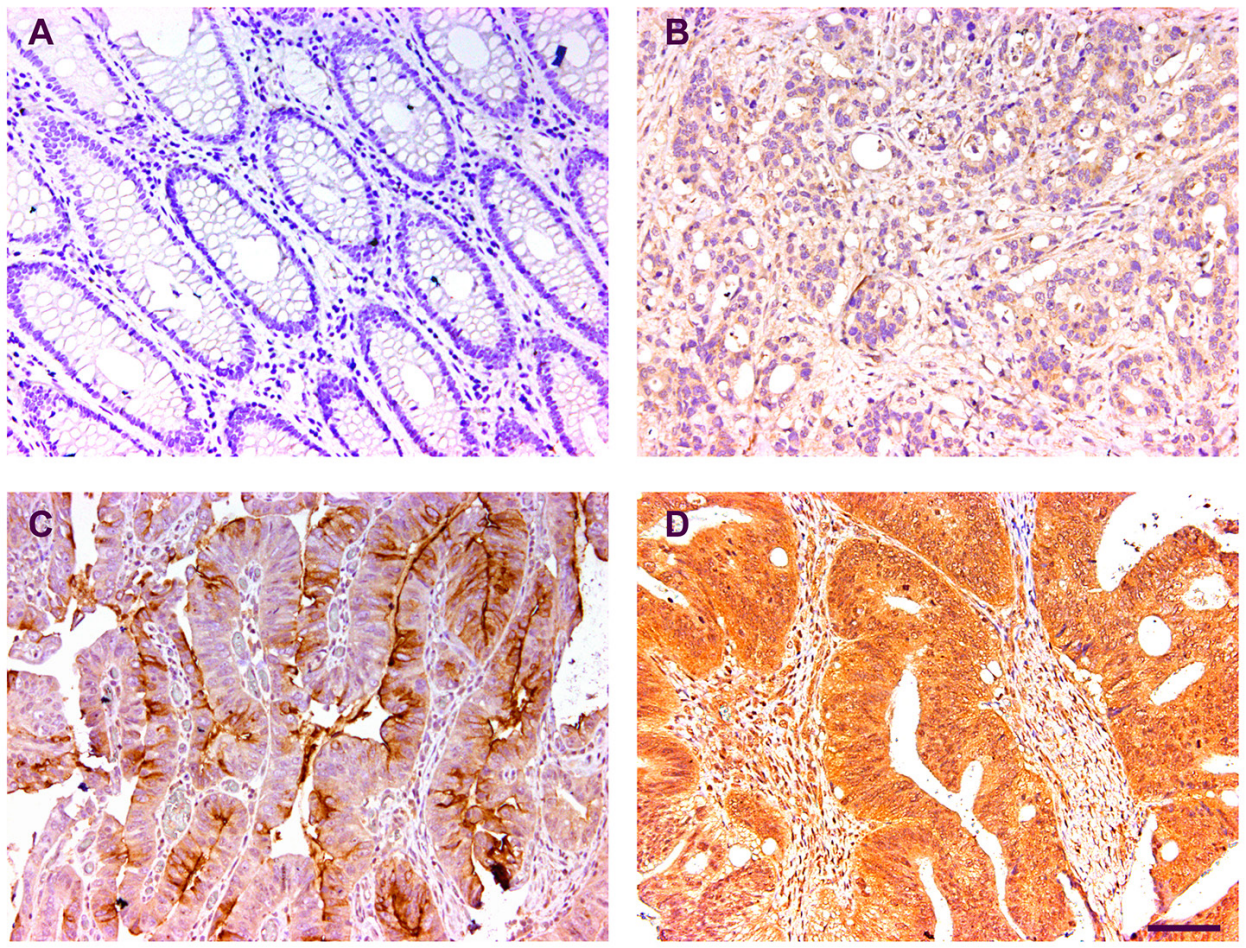
Representative immunohistochemical images of the CST1 expression **(A)** Negative control (normal tissue). **(B-D)** Representative photographs of weak, moderate and strong staining. Bar, 100 μm.

To assess the expression of CST1 in CRC, we examined CST1 expression in 375 cases of CRCs and matched nontumor tissues by IHC. After identification of CRCs using hematoxylin-eosin staining, immunoreactivity of the CST1 protein was observed in the cytoplasm (Figure 1). CST1 expression in CRC tissues was apparently higher than that in nontumor tissues (*P* < 0.001, Supplementary Table 1), suggesting that CST1 in involved in CRC tumorigenesis. We investigated the relationship between CST1 expression and the clinicopathological characteristics in 375 CRC patients, as listed in Table 1. Results demonstrated that no significant correlation existed between CST1 expression and clinical pathological characteristics (Table 1).

**Table 1:** Clinical characteristics of patients according to CST1 in the training and validation cohorts

Variables	Training cohort (n = 234)				Validation cohort (n =141)			
	N	low CST1 (%)	high CST1 (%)	p value	N	low CST1 (%)	high CST1 (%)	p value
**Gender**				0.441				0.655
Male	141	80(56.7%)	61(43.3%)		72	38(52.8%)	34(47.2%)	
Female	93	48(51.6%)	45(48.4%)		69	39(56.5%)	30(43.5%)	
**Age(years)**				0.081				0.077
60	120	59(49.2%)	61(50.8%)		53	34(64.2%)	19(35.8%)	
≧60	114	69(60.5%)	45(39.5%)		88	43(48.9%)	45(51.1%)	
**Tumor location**				0.661				0.093
Colon	131	70(53.4%)	61(46.6%)		77	47(61.0%)	30(39.0%)	
Rectum	103	58(56.3%)	45(43.7%)		64	30(46.9%)	34(53.1%)	
**Differentiation status**				0.047				0.487
Well	84	55(64.3%)	30(35.7%)		63	35(55.6%)	28(44.4%)	
Moderate	118	61(51.7%)	57(48.3%)		59	34(57.6%)	25(42.4%)	
Poor and undifferentiated	32	13(40.6%)	19(59.4%)		19	8(42.1%)	11(57.9%)	
**CEA**				0.251				0.686
Elevated	60	29(48.3%)	31(51.7%)		46	24(52.2%)	22(47.8%)	
Nomal	174	99(56.9%)	75(43.1%)		95	53(55.3%)	42(44.2%)	
**CA199**				0.315				0.840
Elevated	40	19(56.2%)	21(43.8%)		23	64(54.2%)	54(45.8%)	
Nomal	194	109(47.5%)	85(52.5%)		118	13(56.5%)	10(43.5%)	
**Depth of invasion**				0.960				0.534
T1	9	5(55.6%)	4(44.4%)		5	4(80.0%)	1(20.0%)	
T2	20	12(60.0%)	8(40.0%)		20	12(60.0%)	8(40.0%)	
T3	167	91(54.5%)	76(45.5%)		94	48(51.1%)	46(48.9%)	
T4	38	20(52.6%)	18(47.4%)		22	13(59.1%)	9(40.9%)	
**Lymph node metastasis**				0.197				0.133
N0	126	71(56.3%)	55(43.7%)		80	49(61.3%)	311(38.7%)	
N1	77	45(58.4%)	32(41.6%)		43	21(48.8%)	22(51.2%)	
N2a	11	3(32.7%)	8(72.7%)		12	6(50.0%)	6(50.0%)	
N2b	20	9(45.0%)	11(55.0%)		6	1(16.7%)	5(83.3%)	
**TNM stage**				0.496				0.168
I	21	14(66.7%)	7(33.3%)		18	12(66.7%)	6(33.3%)	
II	101	55(54.5%)	46(45.5%)		62	37(59.7%)	25(40.3%)	
III	112	59(52.7%)	53(47.3%)		61	28(45.9%)	33(54.1%)	
Adjuvant chemotherapy				0.139				0.430
No	109	54(49.5%)	55(50.5%)		61	31(50.8%)	30(49.2%)	
Yes	125	74(59.2%)	51(40.8%)		80	46(57.5%)	34(42.5%)	
Adjuvant radiotherapy				0.360				0.228
No	212	118(55.7%)	94(44.3%)		125	66(52.8%)	59(47.2%)	
Yes	22	10(45.5%)	12(54.5%)		16	11(68.8%)	5(31.3%)	

CEA: carcino-embryonic antigen.

### High expression of CST1 is associated with poor clinical outcome

**Figure 2:**
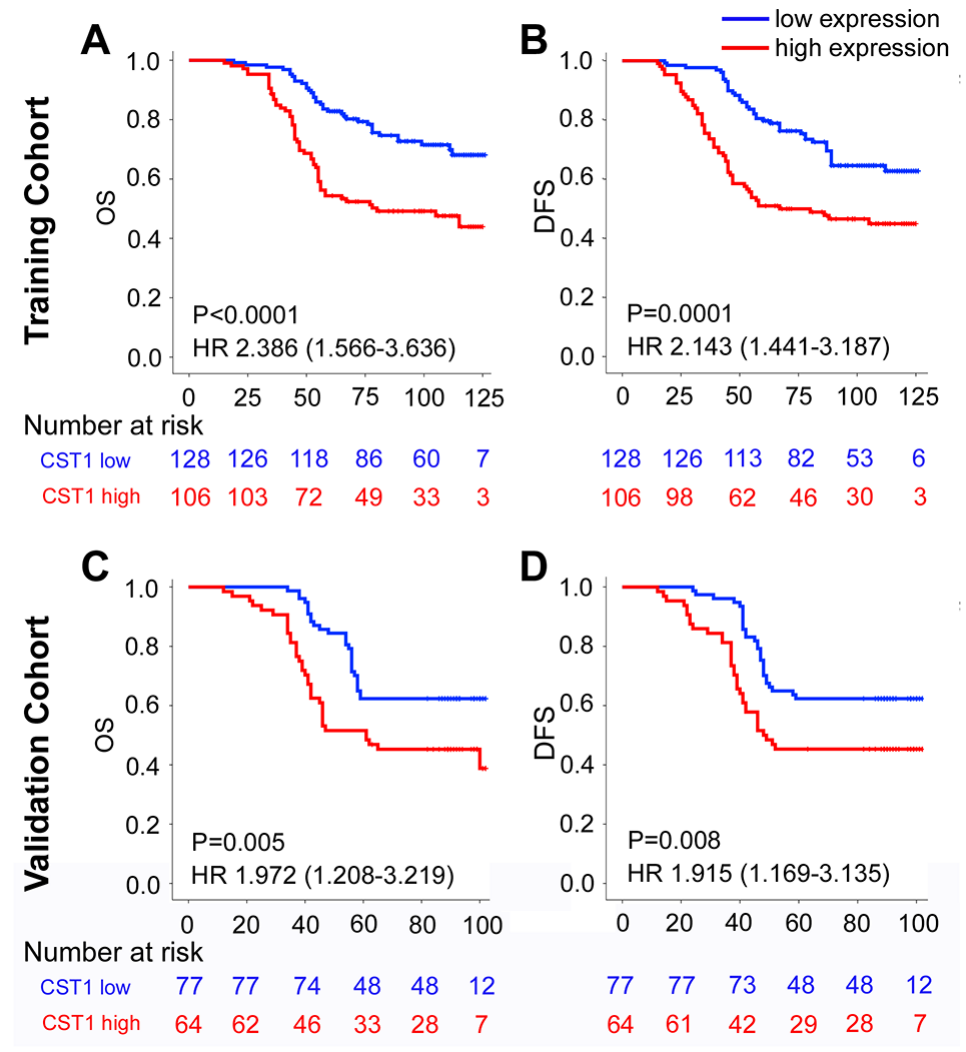
Kaplan-Meier survival analysis of overall survival and disease-free survival according to CST1 expression status of CRC patients in the training cohort and validation cohort The up panel shows the results from the training cohort, and the low panel shows the results from the validation cohort. **(A), (B)** Training Cohort, and **(C), (D)** Validation Cohort. (A), (C) OS, and (B), (D) DFS.

To investigate the prognostic value of CST1 expression for clinical outcomes in CRC patients, Kaplan–Meier survival analysis was performed to compare DFS and OS according to CST1 expression. In the training cohort, the 5-year OS and DFS were 53.8% and 50.9% for patients with CST1^high^ respectively, while were 82.8% and 80.5% for patients with CST1^low^ respectively (Figure. 2A-2B). To confirm that the CST1 expression had a prognostic value in different populations, we further carried the same assay in the validation cohort, and obtained similar results (Figure. 2C-2D). In multivariate analysis using the clinicopathological variables, CST1 was an independent prognostic factor in the prediction of DFS and OS (Table 2, Supplementary Tables 2-4). When stratified by clinicopathological risk factors, CST1 remained a clinically and statistically significant prognostic marker (Supplementary Figure 1-2).

**Table 2:** Multivariable Cox regression analysis of survival in the training cohort

Variables	Overall survival		Disease-free survival	
	HR (95% CI)	*p* value	HR (95% CI)	*p* value
CEA(ng*/*ml) (elevated vs. nomal)	1.874 (1.198-2.932)	0.006	1.784 (1.175-2.710)	0.007
Differentiation status		0.028	/	/
moderate vs. well	1.349 (0.801-2.272)	0.260	/	/
poor vs. well	2.427 (1.280-4.601)	0.007	/	/
Depth of invasion		0.003		<0.0001
T2 vs. T1	0.777 (0.128-4.734)	0.784	1.411 (0.143-13.935)	0.768
T3 vs. T1	1.453 (0.346-6.101)	0.610	3.193 (0.436-23.408)	0.253
T4 vs. T1	3.493 (0.813-14.996)	0.093	8.135 (1.093-60.525)	0.041
Lymph node metastasis		0.0003		0.0003
N1 vs. N0	1.231 (0.708-2.141)	0.462	1.337 (0.798-2.241)	0.270
N2a vs. N0	2.276 (0.992-5.221)	0.052	2.412 (1.117-5.209)	0.025
N2b vs. N0	4.072 (2.153-7.700)	<0.0001	3.909 (2.121-7.202)	<0.0001
CST1 (high vs. low)	2.252 (1.466-3.461)	0.0002	2.118 (1.410-3.182)	0.0003

CEA: carcino-embryonic antigen.

### Development and validation of nomograms for predicting prognosis of CRC patients

**Figure 3:**
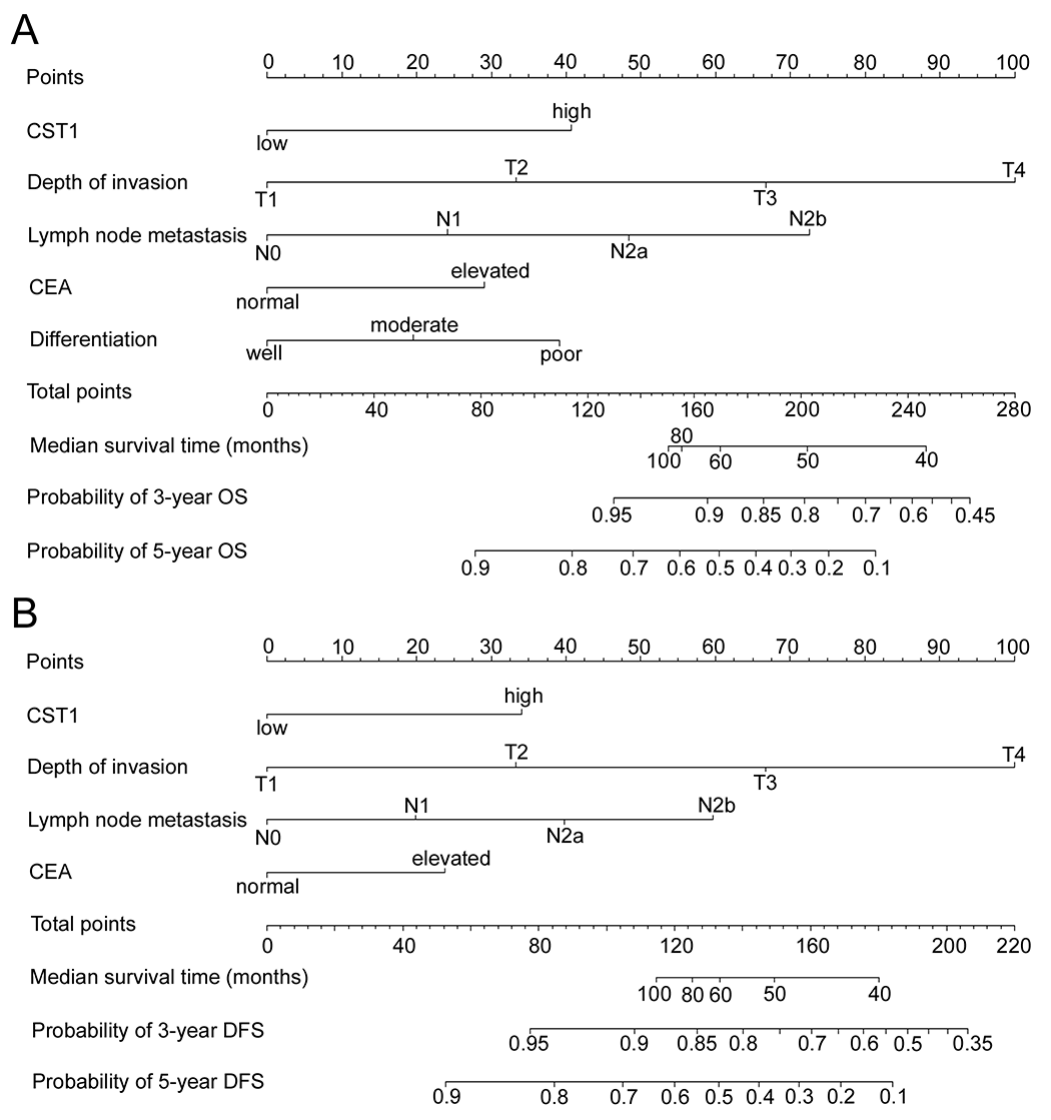
Nomogram for predicting overall survival (OS) and disease-free survival (DFS): Locate the grade of the patient on the grade axis and then draw a straight line upward to the Points axis to determine how many points toward survival the patient receives for her/his grade Repeat this process for the other axes, each time drawing a straight line upward toward the Points axis. Take the sum of the points received for each predictor and locate this sum on the Total Points axis. Draw a straight line down to the survival-probability axis to find the patient’s probability of surviving colorectal cancer. **(A)** OS nomogram, and **(B)** DFS nomogram.

**Figure 4:**
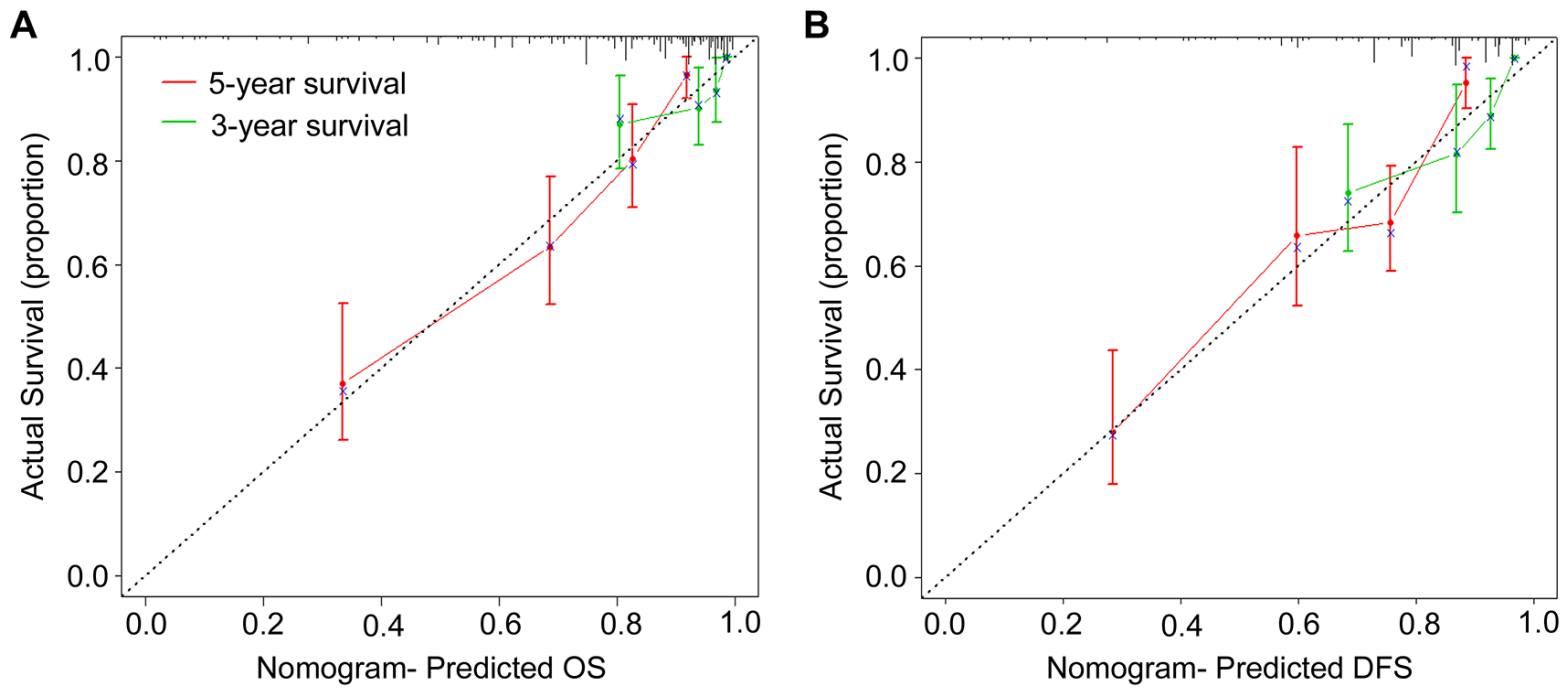
Calibration curves for the nomogram The calibration curve for predicting patient OS **(A)** and DFS **(B)** at 3- and 5-year in the training cohort. Nomogram-predicted OS and DFS are plotted on the x-axis, and actual OS and DFS are plotted on the y-axis. The dotted line represents an ideal nomogram, and the solid blue line represents the current nomogram. The vertical bars are 95% CIs, and the ×’s are bootstrap-corrected estimates.

To predict OS and DFS of CRC patients, two nomograms were established by multivariate Cox regression model according to all significantly independent factors for OS and DFS (Figure. 3A-3B). Nomograms can be interpreted by summing up the points assigned to each variable, which is indicated at the top of scale. The total points can be converted to predicted 3- and 5-year OS and DFS for a patient in the lowest scale (28). In the training cohort, the C-indexes for OS and DFS prediction were 0.767 (95% CI: 0.723-0.811) and 0.743 (95% CI: 0.700-0.786), respectively (Supplementary Table 5). Calibration curves for two nomograms (Figure 4) revealed no deviations from the reference line and no need of recalibration. In the validation cohort, the C-indexes for OS and DFS prediction were 0.741 (95% CI: 0.680-0.802) and 0.717 (95% CI: 0.655-0.779), respectively. The calibration curves yielded agreement between predicted and observed outcomes for OS and DFS (Supplementary Figure 3). Furthermore, the performance of the nomograms for OS and DFS were superior to TNM stage in training cohort and validation cohort (Supplementary Table 5).

**Figure 5:**
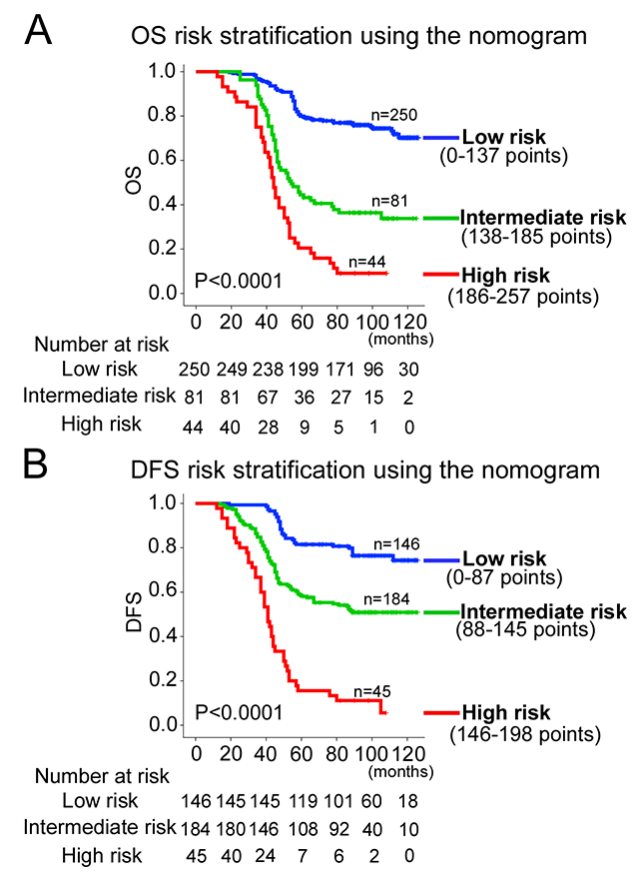
Kaplan-Meier survival analysis of OS and DFS according to three risk groups The entire population was divided in 3 subgroups according to the total number of points given by the nomograms. **(A)** OS nomogram; and **(B)** DFS nomogram.

Using the X-title, the composite scoring was divided to three risk groups accurately discriminated patients with good, intermediate, and poor prognosis (Figure 5 and Supplementary Figure 4). Therefore, we further made subgroup analysis in stage II and III CRC patients respectively. The three risk groups can significantly distinguish patients from different prognosis in stage II or III CRC patients (Supplementary Figures 5-6).

### Clinical usage

**Figure 6:**
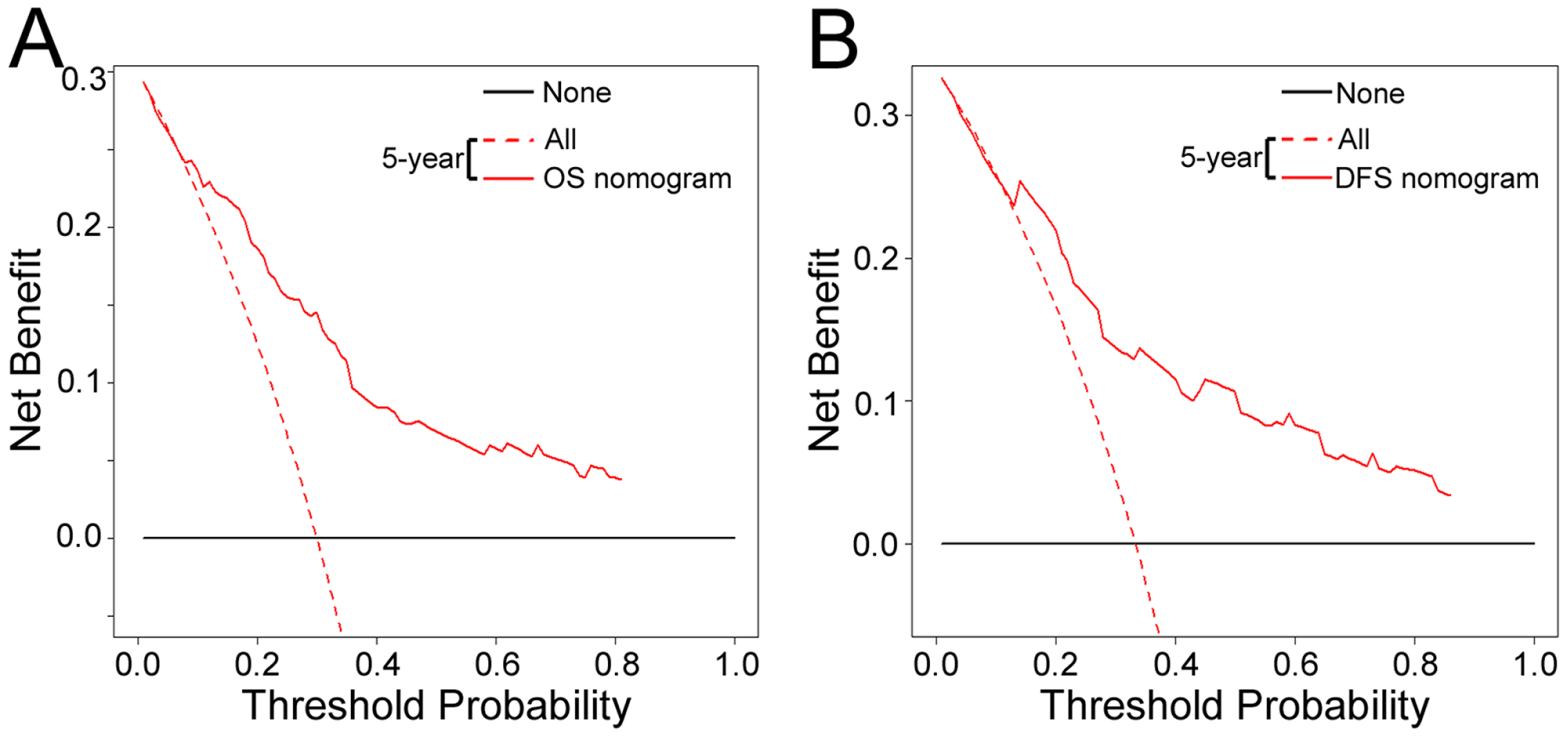
Decision curve analysis for the two nomograms in the training cohort The y-axis measures the net benefit. The solid blue and red line represents the nomogram. The dotted blue and red line represents the assumption that all patients have 5-year survival. Thin black line represents the assumption that no patients have 5-year survival. The net benefit was calculated by subtracting the proportion of all patients who are false positive from the proportion who are true positive, weighting by the relative harm of forgoing treatment compared with the negative consequences of an unnecessary treatment. Here, the relative harm was calculated by [pt/(1 – pt)]. “pt” (threshold probability) is where the expected benefit of treatment is equal to the expected benefit of avoiding treatment; at which time a patient will opt for treatment informs us of how a patient weighs the relative harms of false-positive results and false-negative results ([a - c]/[b - d] = [1 - pt]/pt); a - c is the harm from a false-negative result b - d; is the harm from a false-positive result. a, b, c and d give, respectively, the value of true positive, false positive, false negative, and true negative. The decision curve showed that if the threshold probability of a patient or doctor is > 10%, using the nomogram in the current study to predict 5-year survival adds more benefit than the treat-all-patients scheme or the treat-none scheme. **(A)** OS nomogram, and **(B)** DFS nomogram.

The decision curve analysis for the two nomograms is presented in Figure 6 and Supplementary Figure 7. The decision curve showed that if the threshold probability of a patient or doctor is > 10%, using the two nomograms to predict 5-year OS and DFS adds more benefit than either the treat-all-patients scheme or the treat-none scheme. Within this range, net benefit was comparable, with several overlaps, on the basis of the nomograms.

## DISCUSSION

Albeit increasing evidence showed roles of CST1 in tumor invasion and metastasis, its clinical and prognostic significance in CRC patients has not been well established. In this study, we have demonstrated that the CST1 expression is closely associated with survival and is an independent prognostic parameter for OS and DFS in CRC patients. Based on CST1 expression and four clinicopathological risk variables, two nomograms were generated and validated for predicting 3- and 5-year OS and DFS probabilities after curative resection. Good discrimination and calibration provide this model as a simple and easy tool for predicting the survival of individual Chinese CRC patients.

CST1 protein contains 121 amino acids and acts as an active cysteine protease inhibitor for CST superfamily. Previous studies have already indicated that CST1 is a potential biomarker for human cancers. The mRNA level of CST1 is increased in gastric cancer tissues and is closely linked to the pTNM stage [21]. In addition, overexpression of exogenous CST1 promotes AGS cell proliferation, while knockdown of CST1 plays an opposite role [21]. High expression of CST1 is demonstrated to be a signal of higher rate of recurrence, metastatic risk, and poor survival in patients with surgically resected NSCLCs [23]. Furthermore, CST1 also contributes to the proliferation of pancreatic cancer cells and acts a potential biomarker for the early detection of pancreatic cancers [22]. Perplexingly, overexpression of CST1 apparently improves survival of patients with surgically resected ESCC, which displays a reverse effect compared with other types of tumors. This contradiction suggests that CST1 possibly plays oncogenic or anti-tumor roles in distinct cancers [24]. The previous studies showed that CST1 mRNA and protein are upregulated in CRC cell lines and tissues. In addition, CST1 protein is increased in the serum and urine of patients with CRC compared with healthy controls. Kim et al. demonstrates that the upregulation of CST1 in CRC contributes to colorectal tumorigenesis by neutralizing the inhibitory effect of CST3 on cathepsin B’s proteolytic activity. These findings suggest that CST1 expression might provide important information regarding the prognosis of CRC patients. However, before this study, there was no evidence that demonstrated the prognosis value of CST1 in CRCs. Our study figured out that CST1^high^ patients had poorer OS and DFS compared with CST1^low^ patients, and also demonstrated that the levels of CST1 protein in CRCs after radical surgery can serve as an independent predictor of patient outcomes.

The TNM stage is the most commonly used system to predict survival for patients who have undergone curative resection for CRC. However, CRC patients within the same stage have different cellular, genetic, and clinicopathological characteristics, and their survival is not uniform, indicating the TNM stage may be insufficient to distinguish CRC patients’ survival. To provide a more individualized staging system, nomograms have been developed to evaluate a lot of significant clinicopathologic predictors to better predict the prognosis of individual patients. Improved prediction of individual outcomes would be applied to counsel patients, individualize treatment, and schedule patients’ follow-ups [26, 27]. In this study, we developed and validated two nomograms including CST1 expression, T stage, N stage, the level of CEA, and differentiation to improve the accuracy of prognosis prediction for CRC patients. These nomograms can be used to better predict an individual patient’s probability of 3- and 5-year OS and DFS. Validation of the nomograms was performed using calibration plots and the C-index. The nomograms performed well with a good calibration. Furthermore, the C-index for OS and DFS was satisfactory (0.767 (0.723-0.811), 0.743 (0.700-0.786), respectively in the training cohort), which was superior to TNM stage (C-index: OS, 0.647 (0.599-0.696); DFS, 0.630 (0.583-0.677); in the training cohort; Supplementary Table 5).

Moreover, the improved survival estimates calculated using the nomograms may assist in identifying patients with a high risk of poor clinical outcome within known TNM stages, as well as in facilitating the choice of therapies. Current guidelines recommend adjuvant chemotherapy for high-risk patients with stage II CRC. The risk of recurrence or poor outcomes in stage II disease has been clinically identified based on the following: fewer than 12 lymph nodes analyzed after surgery, poorly differentiated histology (excluding those with MSI-H), lymphatic/vascular invasion, perineural invasion, bowel obstruction, localized perforation, and close, indeterminate, or positive margins [28]. However, these clinicopathological risk factors do not clearly identify the high-risk patients who are likely to benefit from additional treatments after surgery [4]. Recently, cathepsins have been shown to be part of the dynamic response to anticancer therapy within the tumor microenvironment, and they play essential roles in the development of therapeutic resistance [29, 30]. In a RIP1-Tag2 mouse model of pancreatic islet cell tumorigenesis, Bell-McGuinn et al. showed that cysteine cathepsin inhibition in combination with two distinct regimens of chemotherapy administration (maximum tolerated dose or chemo-switch) resulted in tumor regression, decreased tumor invasiveness, and increased survival [31]. Combining with the association of CST1 expression with malignant behaviors, including proliferation and metastasis, we could raise the hypothesis that patients with high CST1 expression might more likely benefit from cytotoxic chemotherapy. Hence, the two nomograms, which incorporate the status of CST1 expression into the current staging system, might contribute to select patients with poor odds of survival who could benefit from adjuvant chemotherapy. Furthermore, ongoing experiments in preclinical models of various tumor types should pay attention to the general therapeutic feasibility of cathepsin inhibition, alone and in combination with chemotherapy. These results encourage the development and continuing assessment of cysteine cathepsin inhibitors as cancer therapeutics and the exploration of further molecular mechanisms of cystatin involved tumorigenesis and chemotherapeutic response.

In colorectal cancer, the immunoscore may add to the significance of the current AJCC/UICC TNM classification, since it has been demonstrated to be a prognostic factor superior to the AJCC/UICC TNM classification [32–35]. However, there were not studies about the relationship of CST1 with tumor infiltrating lymphocytes. In the future, we will integrate the markers of tumor infiltrating lymphocytes into the nomograms, that may improve the accuracy of the nomograms. Additionally, our study is a retrospective study that relied exclusively on a single-institutional database. Validation by external cohorts is required for the prognosis value of CST1 and the generalized use of the nomograms as the basis for postoperative treatment recommendations. Clearly, our results should be further validated by prospective studies in multicenter clinical trials.

Collectively, this is the first study to show that CRC patients high CST1 protein expression had a higher risk of recurrence/metastasis and poorer survival compared with patients with low CST1 expression. Our findings demonstrate that the levels of CST1 protein expression in CRC after radical surgery can serve as an independent predictor of patient outcomes. The two nomograms were constructed and validated for predicting the probability of 3- and 5-year OS and DFS after curative resection. The nomograms performed well with good discrimination and calibration, which suggests that this model is a simple and easy tool for estimating the individualized survival of Chinese patients with CRC. The model may be useful to both clinicians and patients for counseling and decision-making regarding individualized adjuvant treatments as well as follow-up scheduling.

## MATERIALS AND METHODS

### Patients and tissue specimens

We used total 375 formalin-fixed paraffin-embedded (FFPE) specimens from 375 CRC patients in this study. For the training cohort, data were obtained from 234 patients with incident, primary, biopsy-confirmed CRC diagnosed from June 2005 to April 2007 at the first affiliated Hospital of Nanchang University, Nanchang, China. Inclusion criteria were availability of hematoxylin and eosin slides with invasive tumor components, availability of follow-up data and clinicopathological characteristics, no history of treated cancer, and appropriate patient informed consent. In addition, we included an additional 141 patients in internal validation cohort, with the same criteria as above, from April 2007 to April 2008. TNM staging was reclassified according to the AJCC staging manual (seventh edition). All participants were Han Chinese (self-reported). Two independent pathologists reassessed all these samples. The institutional review board of the first affiliated hospital of nanchang university approved the retrospective analysis of anonymous data.

### Immunohistochemistry (IHC)

IHC was carried according to the standard protocol [36, 37]. In brief, tissues were fixed in 10% buffered formalin and embedded in paraffin. Sections were cut in 4 mm, de-waxed in xylene, and rehydrated with graded alcohol solutions. Prior to staining, sections were subjected to blocking with endogenous peroxidase in 1% H_2_O_2_ solution in methanol for 10 min and then microwave heated for 30 min in 10 mM citrate buffer, pH 6.0. Serum blocking was performed using 10% normal rabbit serum for 30 min. The slides were incubated with anti-CST1 antibody at a dilution 1:200 (NBP1-55995, Novus, Littleton, USA) overnight at 4°C and thereafter incubated with an amplification system with a labeled polymer/HRP, EnVision™, (DakoCytomation, Denmark) for 30 min. To visualize the sites of bound peroxidase 0.05% 3, 3´-diaminobenzidine tetrahydrochloride (DAB) was used before counterstaining with a modified Harris hematoxylin.

### Evaluation of IHC staining

Two investigators who were blinded to the clinicopathological data independently evaluated CST1 staining by light microscopy. In this study, at least 300 epithelial cells were counted for each tissue. To ensure the consistency of the scores, discordant cases were reviewed.

The CST1 staining was graded by light microscopy to generate an immunoreactivity score (IRS) [38, 39]. The IRS of CST1 expression was calculated by multiplying the intensity by the extent score. The staining intensity was scored as 0 (negative), 1 (weak, light yellow), 2 (intermediate, yellow-brown), or 3 (strong, brown), and the percentage of positively stained cells was evaluated as 0 (0%), 1 (1%–10%), 2 (11%–50%), 3 (51%–70%) and 4 (71%–100%). The IRS was classified as - (0, negative), 1+ (range from 1 to 4, weak), 2+ (range from 5 to 8, intermediate), or 3+ (range from 9 to 12, strong). We defined - to 1+ as “CST1- low expression” and 2+ to 3+ as “CST1- high expression”.

### Construction of the nomograms

In the training cohort, survival curves for different variable values were generated using the Kaplan-Meier estimates and were compared using the log-rank test. Variables that achieved significance at *P* < 0.05 were entered into the multivariable analyses via the Cox regression model. Statistical analyses to identify independent prognostic factors were performed using SPSS 17.0 (SPSS, Chicago, IL). On the basis of the results of the multivariable analysis, two nomograms were formulated by R 3.0.1 (http://www.r-project.org) with the survival and rms package. Backward step-wise selection was applied by using the likelihood ratio test with Akaike’s information criterion as the stopping rule [40].

### Validation and calibration of the nomograms

The performance of the developed nomograms was tested in the validation cohort. The model performance for predicting outcome was evaluated by calculating the concordance index (C-index) [41]. The value of the C-index ranges from 0.5 to 1.0, with 0.5 indicating a random chance and 1.0 indicating a perfect ability to correctly discriminate the outcome with the model. Calibration of the nomogram for 3- and 5-year OS and DFS was performed by comparing the predicted survival with the observed survival after bias correction.

### Clinical use

Decision curve analysis was carried out to determine the clinical usefulness of the nomograms by quantifying the net benefits at different threshold probabilities [42, 43].

### Statistical analysis

We compared two groups using the *t* test for continuous variables and χ^2^; test for categorical variables. The DFS and OS were calculated in months from the date of surgery to the date of regional recurrence or distant metastasis (for DFS) and death or final clinical follow-up (for OS). The Kaplan-Meier method and log-rank test were employed to estimate DFS and OS. A multivariate Cox proportional hazards regression analysis was performed for all variables found to be significant in a univariate analysis. All the other statistical tests were done with R software (version 3.1.0) and SPSS software (version 17.0). Statistical significance was set at 0.05.

## SUPPLEMENTARY MATERIALS FIGURES AND TABLES


