# Supplementary material for: Prognostic significance of cystatin SN associated nomograms in patients with colorectal cancer
Source: Oncotarget. 2017 Dec 8;8(70):115153–63. doi: 10.18632/oncotarget.23041 (PMC5777761; doi:10.18632/oncotarget.23041)
Supplement: Supplementary file 1 [file oncotarget-08-115153-s001.pdf]

Prognostic significance of cystatin SN associated nomograms in patients with colorectal cancer

SUPPLEMENTARY MATERIALS

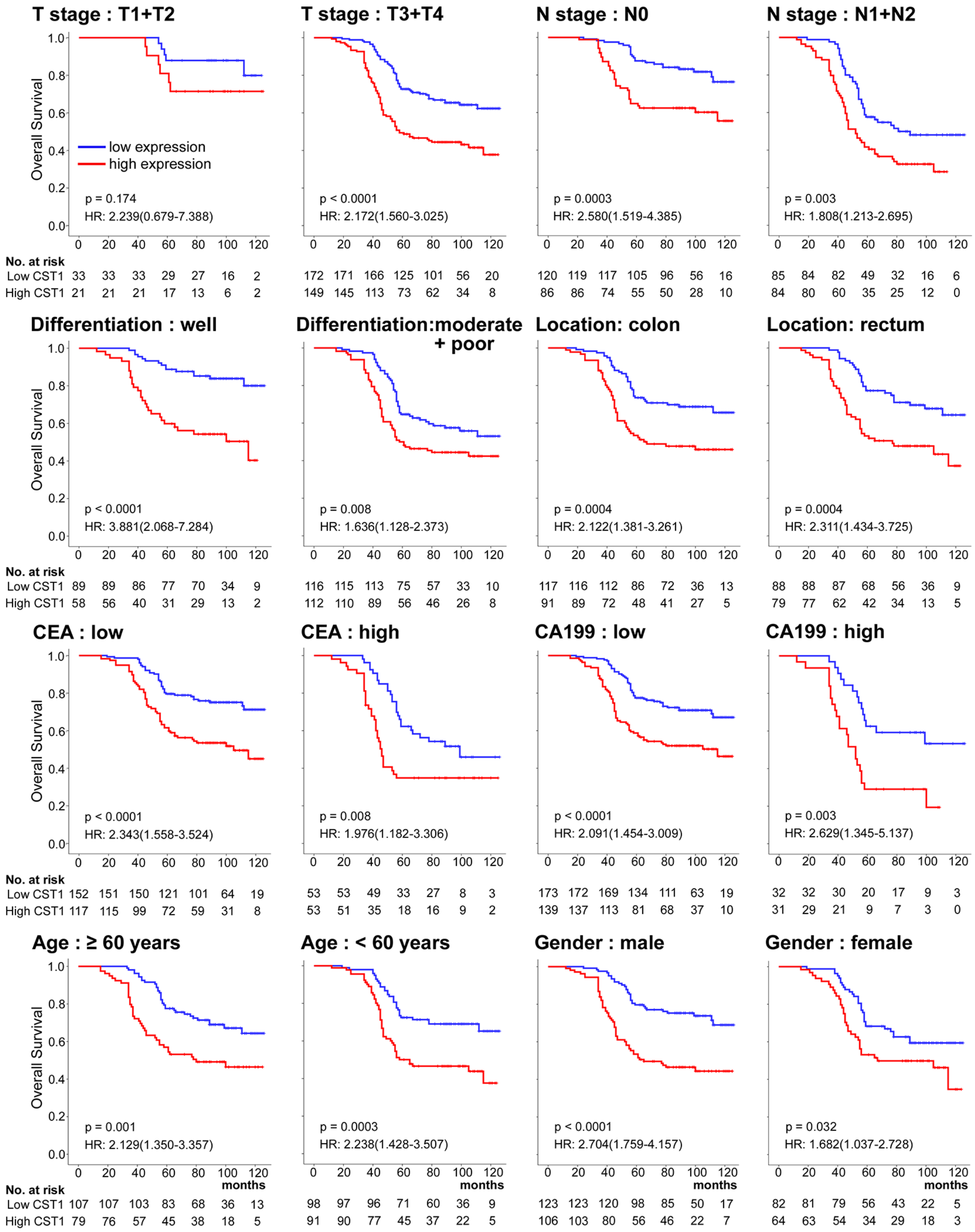

Supplementary Figure 1: Kaplan-Meier survival analysis of OS for all 375 patients with colorectal cancer according to the CST1 expression stratified by clinicopathological risk factors. *P*-values were calculated by log-rank test.

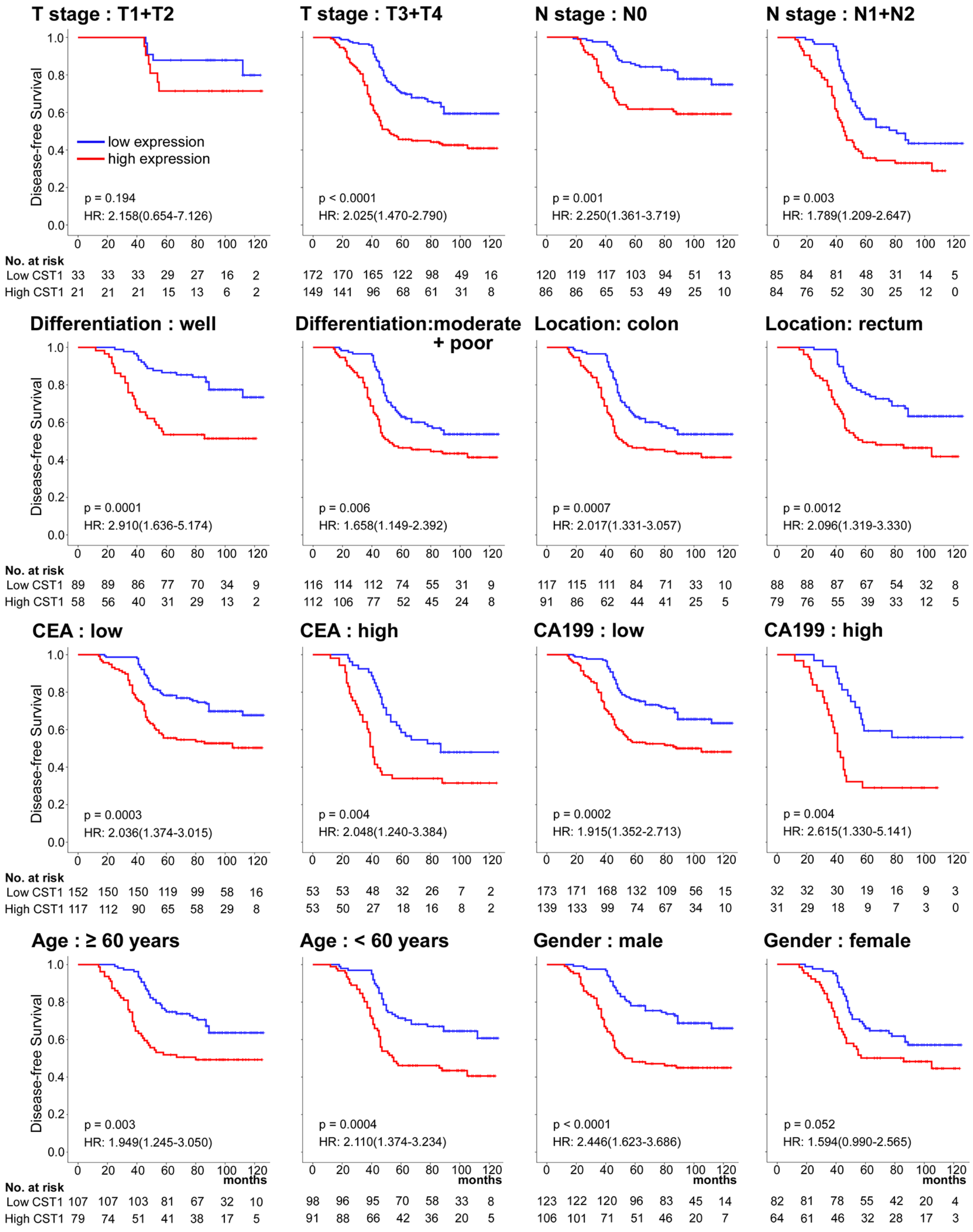

Supplementary Figure 2: Kaplan-Meier survival analysis of DFS for all 375 patients with colorectal cancer according to the CST1 expression stratified by clinicopathological risk factors. *P*-values were calculated by log-rank test.

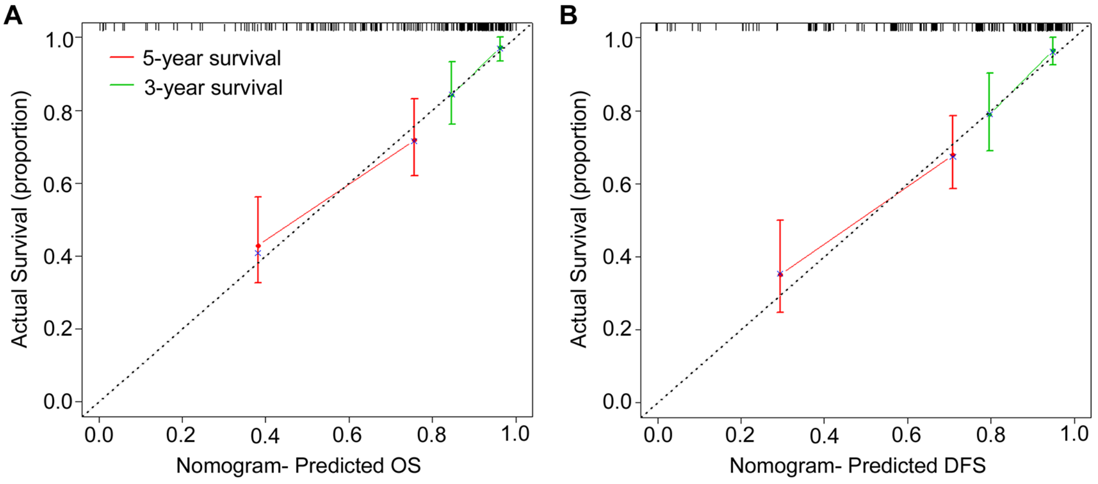

Supplementary Figure 3: Calibration curves for the nomogram in the validation cohort. The calibration curve for predicting patient OS **(A)** and DFS **(B)** at 3-year, 5-years in the validation cohort. Nomogram-predicted OS and DFS are plotted on the x-axis, and the actual OS and DFS are plotted on the y-axis. The dotted line represents an ideal nomogram, and the blue solid line represents the current nomogram. The vertical bars are 95% CIs, and the ×'s are bootstrap-corrected estimates.

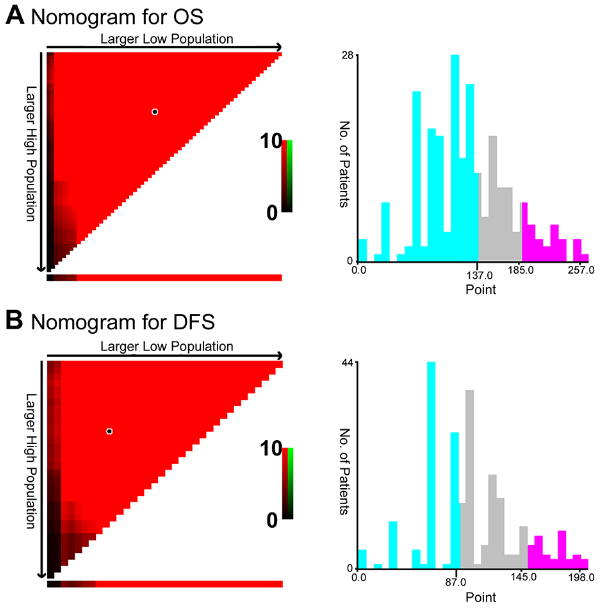

Supplementary Figure 4: X-tile plots of the two nomograms and the points of the nomograms. Coloration of the plot represents the strength of the association at each division ranging from low (dark, black) to high (bright, red, or green). Red represents the inverse association between the expression levels and survival of the feature, whereas green represents a direct association. **(A)** Nomogram for OS , and **(B)** Nomogram for DFS.

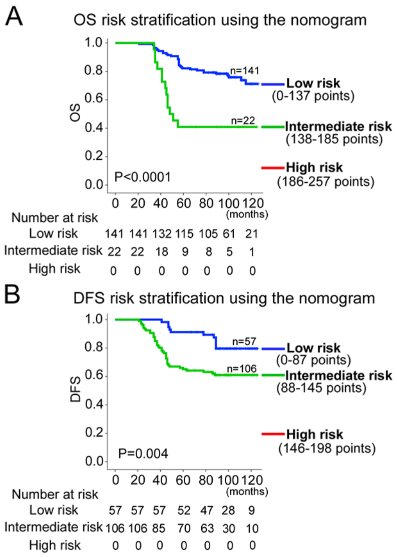

Supplementary Figure 5: Kaplan-Meier survival analysis of OS and DFS according to the three risk groups in stage II CRC patients. The entire population was divided into 3 subgroups according to the total number of points given by the nomograms. **(A)** OS nomogram, and **(B)** DFS nomogram.

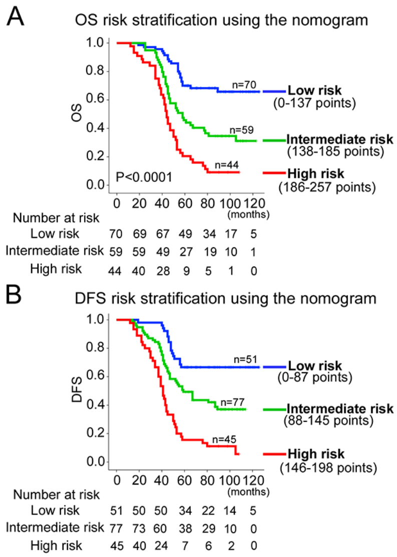

Supplementary Figure 6: Kaplan-Meier survival analysis of OS and DFS according to the three risk groups in stage III CRC patients. The entire population was divided into 3 subgroups according to the total number of points given by the nomograms. **(A)** OS nomogram, and **(B)** DFS nomogram.

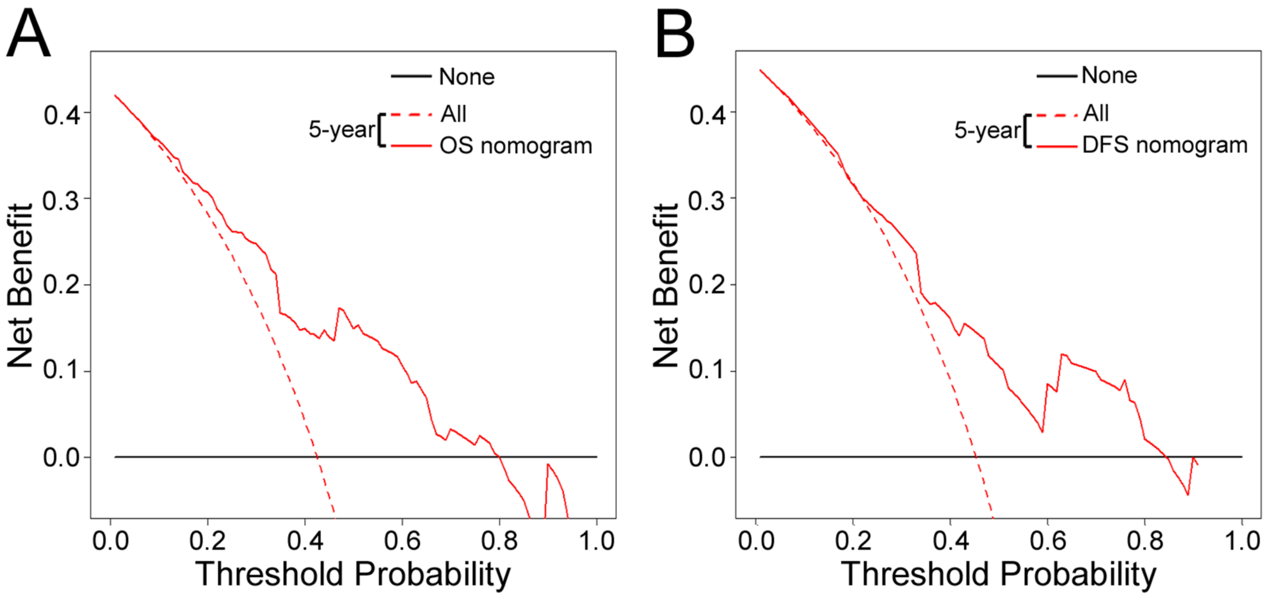

Supplementary Figure 7: Decision curve analysis for the two nomograms in the validation cohort. The y-axis measures the net benefit, and the red line represents the nomogram. The blue line represents the assumption that all patients have 5-year survival, and the thin black line represents the assumption that no patients have 5-year survival. **(A)** OS nomogram, and **(B)** DFS nomogram.

Supplementary Table 1: Immunohistochemistry results for CST1 in CRC compared with normal colorectal tissues in training cohort

CST1*	Tumor tissues (n = 234)	Normal tissues (n = 234)
0	47(20.1%)	88(37.6%)
1+	81(34.6%)	93(39.7%)
2+	64(27.4%)	42(17.9%)
3+	42(17.9%)	11(4.7%)

* χ^2 test $p < 0.0001$.

Supplementary Table 2: Univariate association of disease-free survival in the training cohort

Variables	Training cohort (n = 234)		Validation cohort (n = 141)	
	HR (95%CI)	p value	HR (95%CI)	p value
Age(years) (≥60 vs. <60)	1.327 (0.894-1.970)	0.16	1.090 (0.667-1.779)	0.732
Gender (male vs. female)	1.092 (0.733-1.627)	0.666	1.218 (0.741-2.000)	0.437
Tumor location (colon vs.rectum)	1.098 (0.739-1.632)	0.642	1.281 (0.784-2.092)	0.323
Size(cm) (≥3 vs. <3)	1.071 (0.656-1.749)	0.784	1.704 (0.684-4.246)	0.253
Differentiation status	1.659 (1.231-2.236)	0.001	1.721 (1.221-2.426)	0.002
CEA(ng/ml) (elevated vs. nomal)	2.091 (1.388-3.150)	0.0004	2.005 (1.219-3.297)	0.006
CA199(ng/ml) (elevated vs. nomal)	1.523 (0.940-2.466)	0.087	2.039 (1.142-3.641)	0.016
Depth of invasion	2.918 (2.005-4.246)	<0.0001	1.926 (1.274-2.912)	0.002
Lymph node metastasis	1.818 (1.517-2.178)	<0.0001	1.841 (1.440-2.355)	<0.0001
Adjuvant chemotherapy(yes vs. no)	0.650 (0.438-0.964)	0.032	0.594 (0.364-0.971)	0.038
Adjuvant radiotherapy(yes vs. no)	1.596 (0.890-2.860)	0.116	1.458 (0.719-2.953)	0.296
CST1 (high vs. low)	2.143 (1.441-3.187)	0.0002	1.915 (1.169-3.135)	0.010

Supplementary Table 3: Univariate association of overall survival in the validation cohort

Variables	Training cohort (n = 234)		Validation cohort (n = 141)	
	HR (95%CI)	p value	HR (95%CI)	p value
Age(years) (≥60 vs. <60)	1.260 (0.833-1.905)	0.273	1.122 (0.690-1.827)	0.642
Gender (male vs. female)	1.166 (0.769-1.766)	0.469	1.205 (0.735-1.974)	0.460
Tumor location (colon vs.rectum)	1.091 (0.721-1.650)	0.681	1.212 (0.745-1.972)	0.440
Size(cm) (≥3 vs. <3)	1.158 (0.705-1.902)	0.563	1.803 (0.723-4.498)	0.206
Differentiation status	1.894 (1.387-2.585)	<0.0001	1.756 (1.244-2.478)	0.001
CEA(ng/ml) (elevated vs. nomal)	2.125 (1.384-3.264)	0.001	1.870 (1.140-3.068)	0.013
CA199(ng/ml) (elevated vs. nomal)	1.709 (1.048-2.788)	0.032	2.164 (1.229-3.810)	0.008
Depth of invasion	2.957 (1.997-4.378)	<0.0001	2.009 (1.325-3.045)	0.001
Lymph node metastasis	1.881 (1.562-2.266)	<0.0001	1.877 (1.470-2.397)	<0.0001
Adjuvant chemotherapy(yes vs. no)	0.597 (0.394-0.905)	0.015	0.596 (0.366-0.971)	0.037
Adjuvant radiotherapy(yes vs. no)	1.507 (0.801-2.832)	0.203	1.404 (0.693-2.843)	0.346
CST1 (high vs. low)	2.386 (1.566-3.636)	<0.0001	1.972 (1.208-3.219)	0.007

Supplementary Table 4: Multivariable Cox regression analysis of survival in the validation cohort

Variables	Overall survival		Disease-free survival	
	HR (95% CI)	p value	HR (95% CI)	p value
CEA(ng/ml) (elevated vs. nomal)	2.106 (1.248-3.555)	0.005	2.225 (1.319-3.752)	0.003
Depth of invasion		0.020		0.061
T2 vs. T1	0.589 (0.066-5.226)	0.635	0.580 (0.065-5.157)	0.625
T3 vs. T1	1.145 (0.150-8.719)	0.896	1.083 (0.142-8.255)	0.939
T4 vs. T1	2.660 (0.330-21.418)	0.358	2.165 (0.267-17.566)	0.469
Lymph node metastasis		0.0003		0.0003
N1 vs. N0	2.716 (1.517-4.861)	0.001	2.733 (1.514-4.933)	0.001
N2a vs. N0	3.941 (1.788-8.689)	0.001	3.927 (1.785-8.639)	0.001
N2b vs. N0	4.650 (1.674-12.919)	0.003	4.389 (1.586-12.144)	0.004
CST1 (high vs. low)	1.904 (1.115-3.250)	0.018	1.800 (1.060-3.059)	0.030

CEA: carcino-embryonic antigen.

Supplementary Table 5: Performance of models

	C-index	
	DFS	OS
Training cohort		
Stage	0.630(0.583-0.677)	0.647(0.599-0.696)
Nomogram	0.743(0.700-0.786)	0.767(0.723-0.811)
Validation cohort		
Stage	0.658(0.601-0.714)	0.668(0.613-0.723)
Nomogram	0.717(0.655-0.779)	0.741(0.680-0.802)
